# Automatic registration of urban high-resolution remote sensing images based on characteristic spatial objects

**DOI:** 10.1038/s41598-022-15119-4

**Published:** 2022-08-24

**Authors:** Jun Chen, Zhengyang Yu, Cunjian Yang, Kangquan Yang

**Affiliations:** 1 School of Resources and Environment, Chengdu University of Information Technology, Chengdu, China; 2 Key Laboratory of Land Resources Evaluation and Monitoring in Southwest, Ministry of Education, Sichuan Normal University, Chengdu, China; 3 Sichuan Meteorological Observatory, Chengdu, 610072 China

**Keywords:** Geomorphology, Core processes

## Abstract

Automatic registration of high-resolution remote sensing images (HRRSIs) has always been a severe challenge due to the local deformation caused by different shooting angles and illumination conditions. A new method of characteristic spatial objects (CSOs) extraction and matching is proposed to deal with this difficulty. Firstly, the Mask R-CNN model is utilized to extract the CSOs and their positioning points on the images automatically. Then, an encoding method is provided to encode each object with its nearest adjacent 28 objects according to the object category, relative distance, and relative direction. Furthermore, a code matching algorithm is applied to search the most similar object pairs. Finally, the object pairs need to be filtered by position matching to construct the final control points for automatic image registration. The experimental results demonstrate that the registration success rate of the proposed method reaches 88.6% within a maximum average error of 15 pixels, which is 28.6% higher than that of conventional optimization method based on local feature points. It is reasonable to believe that it has made a beneficial contribution to the automatic registration of HRRSIs more accurately and efficiently.

## Introduction

Image automatic registration technology has a wide range of applications in the fields of computer vision, medical image processing, and remote sensing image processing. To the best of our knowledge, the previous image registration methods mainly include gray level registration^1–5^, transform domain registration^6,7^, and feature-based registration^8,9^. The registration method based on image grayscale is very sensitive to grayscale, rotation, and deformation, but it is not conducive to automatic registration due to the large amount of calculation^10^.

Fourier transform is the most commonly used image registration method in the transform domain. The transformations of image translation, rotation, and affine are reflected in the Fourier transform domain. Note that favorable anti-noise robustness can be obtained by using transform domain method^11^. Nevertheless, its algorithm usually approximates the discrete Fourier transform of points on log polar coordinate grid by interpolation after that on Cartesian grid. Although the algorithm has a small amount of calculation, it has a large interpolation error^12^.

The feature-based registration methods attempt to extract salient features such as edges and corners and use a small amount of local information e.g., correlation of a small image patch^1,13,14^ or local line features^15,16^, to establish matches. The key of the methods is to extract the respective features from two images, and use the spatial relationship of the features to screen out the control points that can be used for registration. Because the feature points are easy to process and analyze, they are applied in the field of image registration widely. Currently, there are many well-known algorithms which have been developed and applied to image registration of remote sensing images, such as Harris^17,18^, SIFT^19–22^, SURF^23–25^, BRISK^26^, ORB^7,27–30^, KAZE^31,32^, and AKAZE^33,34^. With the development of deep learning, several new feature extraction methods have emerged in recent years, mainly including Quad-Net^35^ and SuperPoint^36^. From the point of view of improve invariance property, deep features extracted by artificial neural network are more likely to outperform image gradient-based strategies such as SIFT.

Although image registration methods based on local features have made great progress, local features are still easily affected by local interference. It is difficult to extract relatively consistent features from images of the same region obtained at different times, from different shooting angles and under different lighting conditions, which is the critical bottleneck in the field of image registration of HRRSIs.

In order to achieve image registration of HRRSIs, it is necessary to obtain more stable feature points, which are not easily influenced by local illumination difference, image point displacement, etc. Therefore, the concept of CSO is proposed to try to optimize the image registration algorithm in this paper, and makes a comparative experimental study. Experimental results show that the CSOs in urban HRRSIs has good stability and is less affected by illumination and shooting angle compared with the feature points extracted by conventional methods. This means that it can fulfil the image registration of urban HRRSIs with high success rate and relative high accuracy.

## Methods

**Figure 1:**
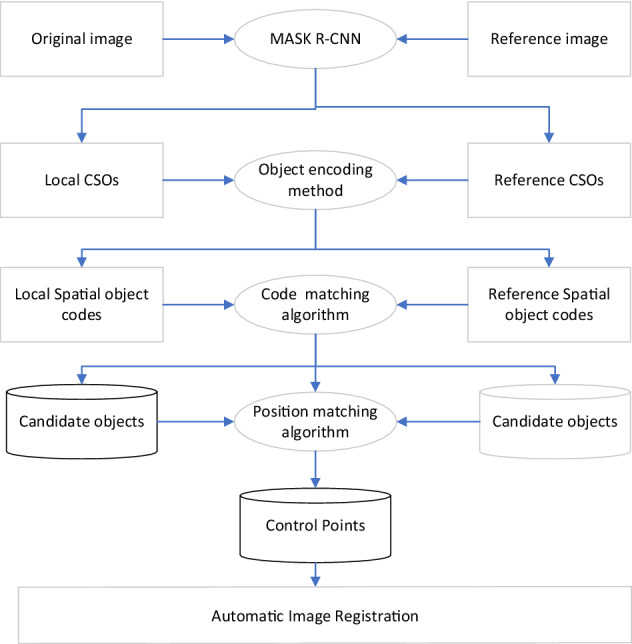
Basic process of image registration based on CSOs.

There are three steps of our method, as shown in Fig. 1. Firstly, we define CSOs in urban HRRSIs and extract them and their positioning point by Mask R-CNN^37,38^. Secondly, encode each of the extracted CSOs according to the category, relative distance, and relative orientation to their 28 neighboring objects. Then, a similarity algorithm of spatial relation code is proposed, and the initial matched object pairs are extracted from the original image and the reference image. Finally, the initial object pairs are further filtered using a position matching algorithm to obtain reliable object pairs. The positioning points of the final object pairs are collected as control points to realize image registration.

### Definition and intelligent extraction of CSOs and their positioning points based on mask R-CNN

#### Definition of CSOs and their positioning points

A characteristic spatial object is one that can be used for automatic image registration. It should have the following characteristics:

1. Identifiable. A spatial object can be automatically and accurately extracted by a computer with existing technology. Therefore, it is necessary to select the objects with relatively stable spectral and morphological characteristics.
2. Locatable. The object has stable location and contains a positioning point on the image. Meanwhile, the pixel displacement is not obvious at different shooting angles.
3. Relatively stable. The object’s spatial location and form remain stable, which ensures the reliability of the object used for image matching.
4. Ubiquitous. The spatial object of selected categories exists widely on the earth's surface. The problem that images cannot be matched due to the lack of objects may be avoided to some extent with this characteristic.

The positioning point of each CSO must be defined for automatic registration of remote sensing images. Generally, the position near the center of a spatial object which is easy to be located can be selected as the positioning point. For example, since the center lines of urban intersections are generally visible on the remote sensing images, the intersection point of the center lines can be defined as the location point of each urban intersection.

#### Intelligent extraction of CSOs and their positioning points based on mask R-CNN

**Figure 2:**
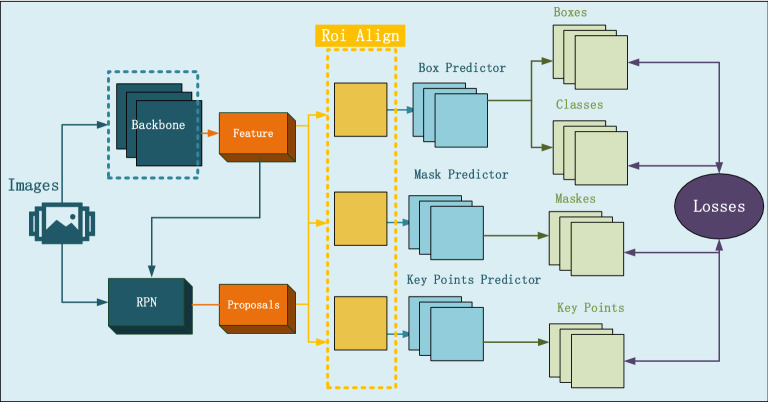
Model structure of Mask R-CNN.

The Mask R-CNN model is used to extract CSOs, which is an extension of Faster R-CNN^39,40^ and an intelligent model for image instance segmentation. The bounding box and category of each object in the image can be predicted with the box predictor. The mask predictor is applied to predict the mask of each object. The key points of each object will be predicted by adding a branch of key point predictor. The model structure of Mask R-CNN is shown in Fig. 2.

**Figure 3:**
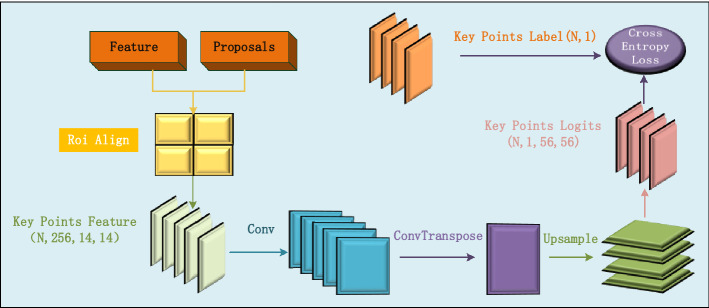
The branch of key point detection used to predict positioning point of each spatial object.

The key points of each object are output through the ROI Align layer and the key points predictor, which are extracted from the features of the object extracted by the backbone and the prediction boxes obtained by the region proposal networks (RPN). In order to extract the positioning point, the number of key points of each CSO is defined as 1 in the branch of key point prediction, as shown in Fig. 3.

### Object encoding method

The key of image registration is to find a certain number of control points from two images. A control point comes from the positioning point of the same CSO between the original image and the reference image. An object encoding method is proposed to encode each CSO of two images, which is used to find the same object pairs.

#### Coding framework

The code of a spatial object consists of location *P*, category *C* and spatial relationship code *R*. The coding framework is expressed as:

1 $$O_{code} = \{ P,C,R\} ,$$

where *P* is the coordinate of the positioning point of each CSO, and is directly recorded by floating-point number. *C* is the category code identified by Mask R-CNN. *R* records the spatial relationship of a certain number of spatial objects adjacent to each CSO, which is the basic for calculating the similarity of CSOs.

#### Spatial relationship encoding

Spatial relationship code should have scale invariance, angle invariance and has a certain degree of robustness in order to realize remote sensing image registration. The neighboring baseline is introduced to measure the distance and orientation between CSOs. The nearest *N* adjacent neighbors are searched and sorted from near to far to construct the neighboring object sequence for each encoding CSO. The ray connecting its positioning point to that of each neighboring object is called the neighboring baseline, and the rotation angle of the neighboring baseline relative to the *X* axis is called the neighboring angle. The distance between their positioning points is called the neighboring distance.

Taking one of the nearest neighbors as the reference, the relative distance and direction of the remaining neighboring objects are measured by the neighboring distance coefficient *ζ* and neighboring baseline deflection angle *ϕ*.

*ζ* is the ratio of the neighboring distance of the remaining neighboring objects to that of the reference neighboring objects.

2 $$\zeta = \frac{{d_{i} }}{{d_{0} }}\;\;\;\;\;\;\;\left( {1 < i < N} \right),$$

where *d*_0_ refers to the neighboring distance of the reference neighboring object, *d*_*i*_ is the neighboring distance of the *i*th remaining neighboring objects, *N* is the number of nearest adjacent objects participating in the construction of spatial relationship code.

**Figure 4:**
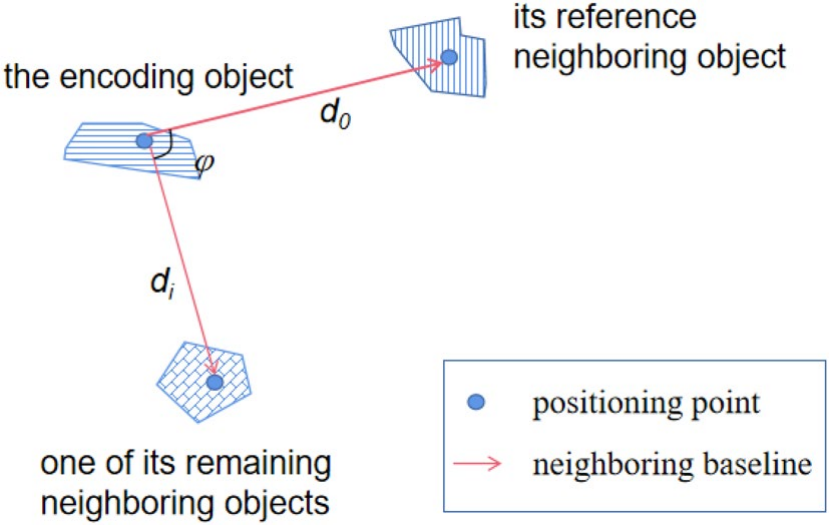
Schematic diagram of the parameters of spatial relationship encoding.

*ϕ* is the deflection angle of the neighboring baseline of the remaining neighboring objects relative to the reference neighboring object.

3 $$\phi = a_{i} - a_{{0}} \;\;\;\;\;\;\;\left( {1 < i < N} \right),$$

where *a*_0_ is the neighboring angle of the reference neighboring object, and *a*_*i*_ is the neighboring angle of the *i*th remaining neighboring objects. The schematic diagram of the parameters in spatial relationship coding is shown in Fig. 4.

The spatial relationship coding rules are as follows:

4 $$F = \left\{ {C_{0} ,\left\{ {C_{i} ,D_{i} ,A_{i} |1 \le i \le N} \right\}} \right\},$$

where *C*0 refers to the category code of the reference neighboring object, and *Ci*, *Di*, and *Ai* represent the category code, distance code and angle code of the remaining neighboring objects, respectively.

Based on the neighboring distance and the neighboring baseline of the reference object, *Di* and *Ai* of remaining neighboring objects are encoded. In order to expand the range of distance code as much as possible and enhance the robustness of spatial relationship code, the stretching value of distance coefficient is calculated as:

5 $$D_{i} = round\left( {\log_{1.15} \zeta } \right),$$

where *Ai* is calculated as follows:

6 $$A_{i} = round\left( {{{\phi^{\prime}} \mathord{\left/ {\vphantom {{\phi^{\prime}} {10}}} \right. \kern-\nulldelimiterspace} {10}}} \right),$$

where *ϕʹ* is:

7 $$\phi^{\prime} = \left\{ \begin{gathered} \phi \;\;\;\;\;\;\;\;\;\;\;\;\;\;\;\;\;0 \le \phi \le 360 \hfill \\ 360 + \phi \;\;\;\;\;\;\;\;\;\phi < 0. \hfill \\ \end{gathered} \right.$$

### Code matching algorithm

#### Calculation of the similarity of spatial relationship codes

The similarity of the spatial relationship codes is used to measure the similarity of two objects. Assume that a spatial object exists in both the object sets of the original image and the reference image, represented as *L* and *S* respectively. Then the code of *L* and *S* are expressed as:

8 $$\left\{ \begin{gathered} F_{L} = \left\{ {C_{0}^{L} ,\left\{ {C_{i}^{L} ,D_{i}^{L} ,A_{i}^{L} \left| {1 \le i < N} \right.} \right\}} \right\} \hfill \\ F_{S} = \left\{ {C_{0}^{S} ,\left\{ {C_{i}^{S} ,D_{i}^{S} ,A_{i}^{S} \left| {1 \le i < N} \right.} \right\}} \right\}, \hfill \\ \end{gathered} \right.$$

where *F*_*L*_ and *F*_*S*_ represent the spatial relationship codes of *L* and *S*, respectively.

The similarity of *F*_*L*_ and *F*_*S*_ is:

9 $$p_{SL} = w\overline{p},$$

where *w* is the matching coefficient and $\overline{p}$ is the average matching degree of the remaining neighboring objects.

Take out one of the remaining neighboring objects from *L* and *S*, respectively, and their similarity is:

10 $$p_{ij} = \left\{ \begin{gathered} \left( {1 - \frac{{\left| {D_{i}^{SL} } \right|}}{5}} \right)\left( {1 - \frac{{\min \left( {\left| {A_{i}^{SL} } \right|,36 - \left| {A_{i}^{SL} } \right|} \right)}}{5}} \right)\;\;\;\;\;\;\;\left( {C_{i}^{S} = C_{i}^{L} } \right) \cap \left( {\left| {D_{i}^{SL} } \right| < 2} \right) \hfill \\ 0\;\;\;\;\;\;\;\;\;\;\;\;\;\;\;\;\;\;\;\;\;\;\;\;\;\;\;\;\;\;\;\;\;\;\;\;\;\;\;\;\;\;\;\;\;\;\;\;\;\;\;\;\;\;\;\;\;\;\;\left( {C_{i}^{S} \ne C_{i}^{L} } \right) \cup \left( {\left| {D_{i}^{SL} } \right| \ge 2} \right), \hfill \\ \end{gathered} \right.$$

where $D_{i}^{SL}$ and $A_{i}^{SL}$ are expressed as

11 $$\begin{gathered} D_{i}^{SL} = D_{i}^{S} - D_{j}^{L} \hfill \\ A_{i}^{SL} = A_{i}^{S} - A_{j}^{L} . \hfill \\ \end{gathered}$$

where *i* and *j* represent one of the remaining neighboring objects of *L* and *S*, respectively.

A certain neighboring object in *L* traverses all the other neighboring objects in *S*, and the similarity is calculated according to Eq. (10). If the maximum similarity is greater than the threshold, it is considered that the neighboring object of *L* has found a match among the neighboring objects of *S*. $\overline{p}$ is calculated by

12 $$\overline{p} = \frac{1}{{N_{SL} }}\sum\limits_{k = 1}^{{N_{SL} }} {p_{k} } \;\;\;\;\;\;\left( {p_{k} \ge \alpha } \right),$$

where *p*_*k*_ is the similarity corresponding to the two matching neighboring objects in *L* and *S*, and *N*_*SL*_ is the total matching number of the remaining neighboring objects; *a* is the similarity threshold.

*w* is calculated as follows:

13 $$w = \left\{ \begin{gathered} \frac{{0.1\left( {N_{SL} { - }\beta } \right)}}{{N{ - }\beta }} + 0.9\;\;\;\;\;\;\;N_{SL} \ge \beta \hfill \\ {0}\;\;\;\;\;\;\;\;\;\;\;\;\;\;\;\;\;\;\;\;\;\;\;\;\;\;\;\;\;N_{SL} < \beta , \hfill \\ \end{gathered} \right.$$

where *β* represents the minimum matching number of neighboring objects required for spatial object matching.

#### Object matching based on spatial relationship codes

As can be seen from above, the selection of reference neighboring object is the key to spatial relationship encoding. Different reference neighboring object will result in completely different spatial relation code for the same CSO.

It cannot be guaranteed that each CSO has the same neighboring objects on two remote sensing images due to the differences of imaging time, image quality and other factors in remote sensing images. To enhance the robustness of the algorithm, *M* nearest neighbor objects are selected from the neighboring object set of each CSO as reference to obtain *M* spatial object codes according to Eq. (4). The similarity of two CSOs is taken as the maximum similarity of these codes.

For each CSO extracted from the original image, the CSO with the greatest similarity is found in the reference image. If the similarity exceeds *a*, a match is considered to be found and put into the initial matched object pairs.

### Position matching algorithm

A position matching algorithm is applied to the initial matched object pairs to acquire the reliable control points. Assume that a certain CSO extracted from the original image is *L*1, and the matched CSO in the reference image is *S*1. Their 28 nearest neighboring objects (or the actual number if less than 28) are searched respectively. Each neighboring object *L*2 of *L*1 is traversed from near to far, to search matched object *S*2 from the nearest neighbor objects of *S*1 through similarity calculation of spatial relationship codes. If *S*2 exists, the rotation angle and scaling factor of the coordinates of the reference image relative to that of original image are calculated as

14 $$\left\{ \begin{gathered} a_{S1} = a_{S1}^{S2} - a_{L1}^{L2} \hfill \\ s_{S1} = {{d_{S1}^{S2} } \mathord{\left/ {\vphantom {{d_{S1}^{S2} } {d_{L1}^{L2} ,}}} \right. \kern-\nulldelimiterspace} {d_{L1}^{L2} ,}} \hfill \\ \end{gathered} \right.$$

where *a*_*S*1_ and *s*_*S*1_ represent the rotation angle and scaling factor, *aL2 L1* and *aS2 S1* are the rotation angles of the neighboring baseline of *L*2 and *S*2 respectively, *dL2 L1* and *dS2 S1* are the neighboring distances of *L*2 and *S*2 respectively.

The coordinate origins of the two images are moved to the locations where *L*1 and *S*1 are located, and then the coordinates of each neighboring object of *L*1 are converted to the coordinate system of the reference image according to Eq. (14) to determine whether there is an object of the same category in the neighboring objects of *S*1 within a certain distance threshold *ε*. If at least 3 neighboring objects of *L*1 satisfy the above condition, *L*1 and *S*1 are considered to be a correct match. From the initial matched object pairs, the correct matches are reserved to construct the reliable object pairs.

### Automatic image registration

The positioning points of the reliable object pairs are collected as control points, which are substituted into the polynomial correction equation to calculate the coordinate transformation from the original image to reference image:

15 $$\left\{ \begin{gathered} x = \sum\limits_{i = 0}^{n} {\sum\limits_{j = 0}^{n - i} {a_{ij} } } u^{i} v^{j} \hfill \\ y = \sum\limits_{i = 0}^{n} {\sum\limits_{j = 0}^{n - i} {b_{ij} } } u^{i} v^{j} , \hfill \\ \end{gathered} \right.$$

where (*x*, *y*) are the coordinates of the original image, (*u*, *v*) are the coordinates of the reference image, *n* is the power of polynomial equation, and the *a*_*ij*_ and *b*_*ij*_ are the undetermined coefficients which are obtained by the least square method.

Generally, the minimum number of control points (*NC*_*min*_) required in image registration is as follws:

16 $$NC_{\min } = {{\left( {n + 1} \right)\left( {n + 2} \right)} \mathord{\left/ {\vphantom {{\left( {n + 1} \right)\left( {n + 2} \right)} 2}} \right. \kern-\nulldelimiterspace} 2}.$$

In particular, at least 3 control points are required when n is set to 1, which is the minimum number of control points to perform registration.

To further obtain reliable control point pairs, the values of *a*_*ij*_ and *b*_*ij*_ in Eq. (15) are evaluated by the least square method, and the predicted coordinates and distance error are calculated for each control point. Then, the control point with the maximum distance error is found. If its error is greater than *ε*, the control point will be removed from the pairs. The current power (n) will be subtracted by 1 if the number of remaining points is less than the minimum number calculated by Eq. (16). Then, the values of *a*_*ij*_ and *b*_*ij*_ are re-estimated with the remaining control points until the maximum distance error is less than *ε* or the number of control points is less than or equals to 3.

After the reliable control points are acquired, the process of automatic image registration is as follows: firstly, the extent of the original image is converted to the output extent according to Eq. (15). Then, a target image is created by the output extent and resolution. Finally, for each pixel of the target image, its coordinates are transformed to the original coordinates according to Eq. (15), to get the pixel value from the original image. The automatic registration is completed when all the pixels of the target image are computed.

## Experiments and results

### Selection and extraction of CSOs and their positioning point

**Figure 5:**
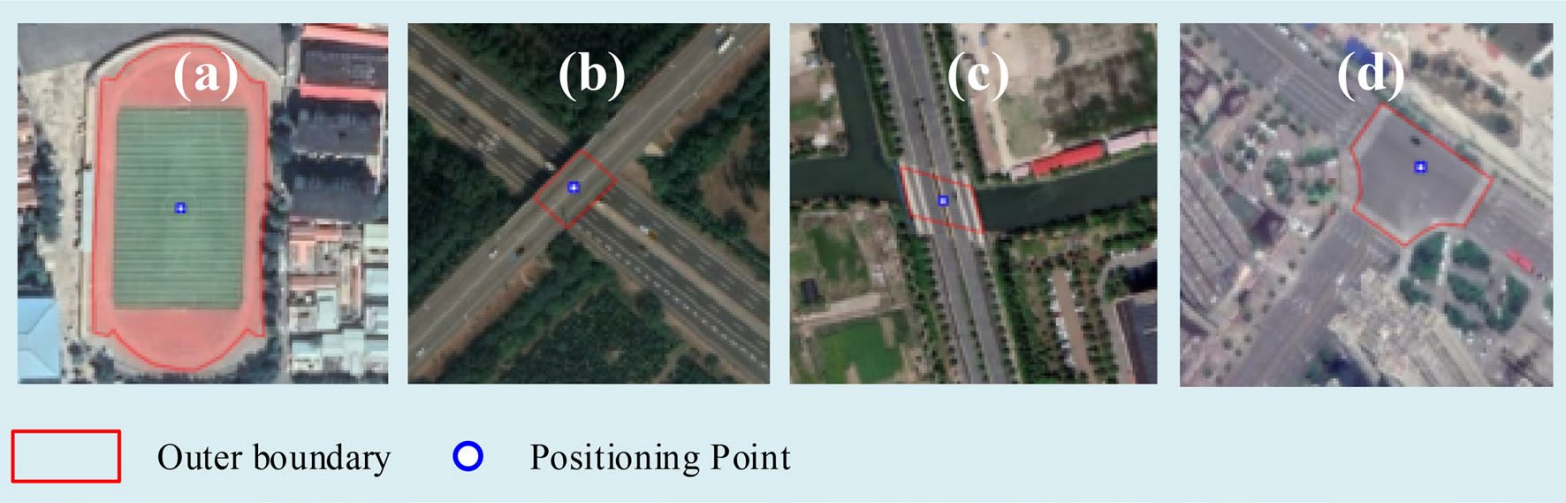
Typical CSOs and their positioning point on from level 18 google online imagery (http://www.google.cn/maps/vt/lyrs=s@167) created by first author (J Chen). (**a**) Sports field, (**b**) across-road bridge, (**c**) across-river bridge and (**d**) urban intersection.

In urban HRRSIs, some artificial objects such as sports fields, across-road bridges, across-river bridges, and urban intersections, have stable morphological and spectral characteristics, which can be extracted by Mask R-CNN easily. Meanwhile, these objects exist widely in the urban area. So, they are selected as CSOs to carry out the experiments of image registration. Figure 5 shows some typical CSOs in urban HRRSIs.

Mask R-CNN is a supervised learning neural network, which requires the preparation of a training data set and a test data set. Each picture in the two data sets represents a local area in the high-resolution remote sensing image. It is necessary to mark all the sports fields, across-road bridges, across-river bridges, and urban intersections in advance for each picture.

Different marking methods are created for different types of CSOs. The regular area enclosed by the outer boundary of the stadium is used to mark a sports field, the center of the stadium is defined as the positioning point. An urban intersection is marked by the area enclosed by the zebra crossings, and the junction of the roads' center lines serves as the positioning point. And for a bridge, it marks the edge of the bridge along the river or road, and their positions can be marked by the center of the isolation belt.

The red lines in Fig. 5 indicate the marking results, and the blue dots indicate their location. There are 1204 images and 1570 labeled objects in the training data set, 200 images and 261 labeled objects in the test data set. Table 1 shows the number of sports fields, urban intersections, and bridges in the two data sets.

**Table 1:** Number of objects and test accuracy of the Mask R-CNN model.

Object	CC	NTRS	NTES	Category		MP (%)	MPE (pixels)
				Recall rate (%)	Precision (%)		
SF	1	372	62	90.32	98.25	84.5	3.5
UI	2	462	76	78.95	93.75	76.4	3.6
AROB	3	389	62	72.58	91.84	83.6	6.3
ARIB	4	347	61	83.61	94.44	82.3	4.1
Total		1570	261	81.23	94.64	81.7	4.3

*SF* sports field, *UI* urban intersection, *AROB* across-road bridge, *ARIB* across-river bridge, *CC* category code, *NTRS* number of training samples, *NTES* number of test samples, *MP* mask precision, *MPE* mean positioning error.

The loss value dropped to a relatively low level and tended to stabilize after 600 epochs of training. It is necessary to set a higher category credibility threshold in order to improve the precision of the model and try to avoid the false detection. The category credibility thresholds of sports fields, urban intersections and bridges are set to be 0.98, 0.98 and 0.97 respectively, with the higher overall precision and relatively high recall rate. The mask threshold is set to 0.5 and the extracted mask is closest to the result of manual discrimination.

**Figure 6:**
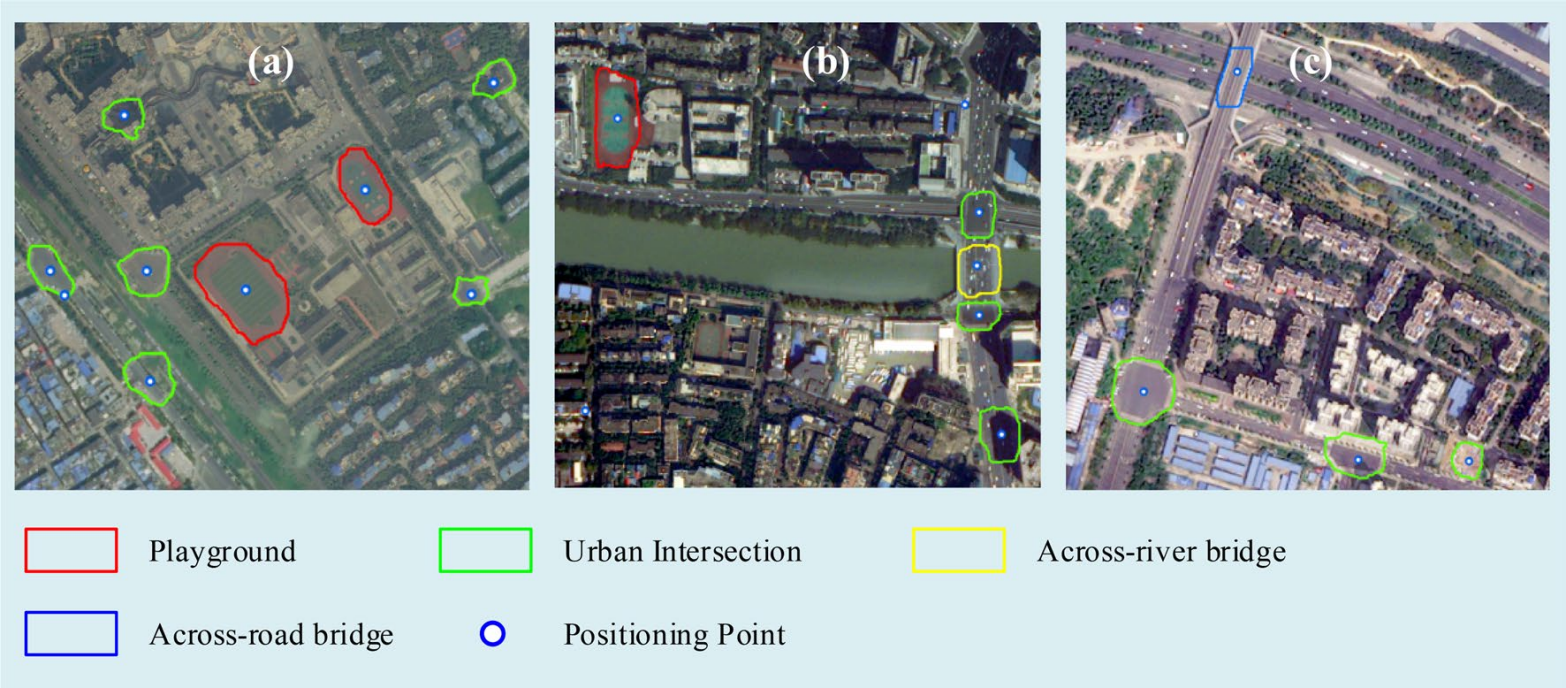
Examples of object extraction from level 18 google online imagery (http://www.google.cn/maps/vt/lyrs=s@167) created by first author (J Chen).

The object extraction results are shown in Table 1. It can be seen that the recall rate is 81.23%, the category precision is 94.64%, and the mask precision is 81.7%. Figure 6 shows the extraction results of three typical sample regions. It can be seen from the figure that the extraction results of Mask R-CNN model are close to human interpretation. On the other hand, there are still a few wrong or missing extractions. For example, the urban intersection in the upper right corner of Fig. 6b is not recognized due to the influence of shadow, and a wrong urban intersection is extracted in the lower right corner of Fig. 6c. Therefore, it requires the image matching algorithm to be fault-tolerant and robust.

### Parameter analysis of spatial relationship encoding and matching

#### Determination of the value of *M*

In order to find a reasonable value of *M* (the number of reference neighboring objects), the area within the first ring road of Chengdu is used as the test area. The 18-level data of Google online satellite imagery and Tianditu online satellite imagery are used to extract the sports fields, across-road bridges, across-river bridges, and urban intersections, and further construct their own object sets. The number of objects extracted by Mask R-CNN model from Google and Tianditu are 822 and 837 in total respectively, as shown in Table 2.

**Table 2:** Number of object pairs with the same neighboring object under different *M* values in the experimental area.

Imagery	NO	NOP	NOPCSNO				
			*M* = 1	*M* = 2	*M* = 3	*M* = 4	*M* = 5
Google	822	491	202	367	438	467	482
Tianditu	837						

*NO* number of objects, *NOP* number of object pairs, *NOPCSNO* number of object pairs containing the same neighboring object.

The two object sets are overlapped and the same two objects are marked as an object pair with the same identifier. The results show that there are 491 identical objects in the above object sets, accounting for 59.8% and 58.7% of the total number of objects, respectively. For each object pair, search the nearest *M* neighboring objects in their object sets. It is believed that the object pair contains the same neighboring object if at least one object pair in their neighboring sequence has the same identifier. Count the number of object pairs with the same neighboring object under different values of *M*, and the results are shown in Table 2.

As shown in Table 2, the number of object pairs with the same nearest object (M = 1) is only 202, accounting for 24.6% and 24.1% of the total number of objects, respectively. The number of object pairs which have same neighbor increases and approaches the true number of object pairs when *M* increases. With the computational efficiency and encoding effectiveness considered, *M* is set to 2 to build to the spatial relationship codes. Therefore, the nearest neighboring spatial relationship code and the second nearest neighboring spatial relationship code are constructed for each CSO.

#### Determination of the value of *N*

The above Tianditu object set and Google object set are continued to be used to find a reasonable value of N, which represents the number of nearest adjacent objects participating in spatial relationship encoding. Traverse each object of Tianditu object set and search for the matched object from Google object set when the value of *α* is set to 0.8 and *β* increases from 7 to 10. If the matched object has the same identifier, add 1 to the number of correct matches. Table 3 shows the correct matches with different value of *N*.

**Table 3:** Correct matches with different number of nearest objects participating in encoding.

N	Correct matches			
	*β* = 7	*β* = 8	*β* = 9	*β* = 10
18	327	301	254	189
20	338	326	311	262
22	338	336	328	307
24	344	342	343	329
26	340	340	343	335
28	344	347	349	345
30	339	345	347	347
32	341	346	350	351
34	341	348	349	350
36	338	346	349	349
60	337	342	348	347

It can be seen from the table that, the number of correct matches first increases with the increase of *N*, and then remains stable or even decreases slightly when *β* is fixed. This means that a certain number of nearest objects are required to construct spatial relationship code in order to find a match. On the other hand, too many nearest objects may have a negative impact on matching to a certain extent because the objects extracted by Mask R-CNN is unreliable. Considering that the larger the value of *β*, the higher the requirements for the number of objects extracted from two images in the actual matching, *β* is set to about 7–10, so *N* is set to 28.

#### Accuracy of object matching algorithm with different values of β

The value of *β* represents the minimum matching number of neighbors required for object matching. Obviously, the greater the value of *β*, the more stringent the object matching requirements. Table 4 shows the matched number with different values of *β* when *α* and *N* are set to 0.8 and 28 respectively.

**Table 4:** Accuracy of object matching with different values of *β* in experimental area.

*β*	TNM	TCM	MA (%)
6	626	334	53.3
8	518	347	70.0
10	439	345	78.6
12	355	285	80.1

*TNM* total number of matches, *TCM* number of correct matches, *MA* matching accuracy.

As can be seen from Table 4, there are only 53.3% of the matched objects are correct when *β*  = 6. The number of total matches decreases and the matching accuracy increases with the increase of *β*. The correct matching rate is over 80% when *β* is greater than or equal to 10. However, the greater *β* is, the larger number of the same objects extracted from two images is needed. Therefore, it is desired to obtain a higher matching accuracy with a smaller *β*.

#### Accuracy of position matching algorithm with different values of *ε*

In Table 4, the matching accuracy is relatively low if only the object matching algorithm is applied. To increase the reliability of the matched pairs, further filtering using the position matching algorithm is required.

Table 5 depicts the correct rate after position matching in experimental area under different values of *ε* and *β* when a is set to 0.8. The first column in the table represents the different values of *ε*, which is a multiple of the cell size of the reference image. As can be seen from the figure, with the increase of distance tolerance, the total matches and the correct matches increase at the same time, but the matching accuracy decreases. It's desired to increase the total number of matches while maintaining a relatively high correct matching rate. Therefore, the distance tolerance (*ε*) is set to 40 times of the pixel resolution of the reference image and the matching accuracy is more than 98%.

**Table 5:** Accuracy of position matching with different values of *ε* in experimental area.

*ε*	Total matched number				Matching accuracy (%)			
	*β * = 6	*β* = 8	*β* = 10	*β* = 12	*β* = 6	*β* = 8	*β* = 10	*β* = 12
10	217	222	216	215	100.0	100.0	100.0	100.0
20	298	304	298	297	100.0	100.0	100.0	100.0
30	323	332	326	322	99.7	100.0	100.0	100.0
40	332	341	335	328	98.5	99.7	99.7	100.0

**Figure 7:**
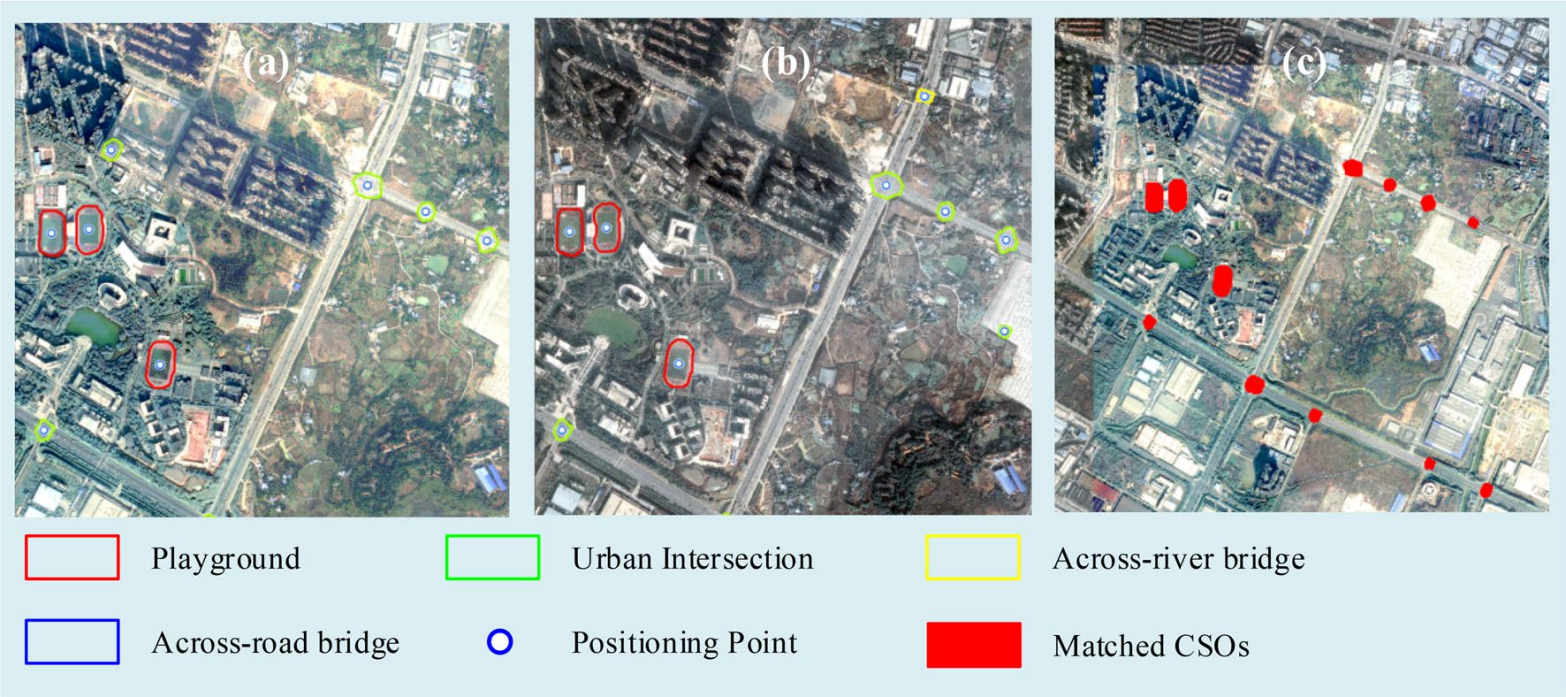
A typical registration result using our method: (**a**) extracted CSOs from original image cropped from level 18 online Tianditu imagery (http://t0.tianditu.gov.cn/img_w/wmts) created by first author (J Chen), (**b**) extracted CSOs from Reference image cropped from level 18 online Google imagery (http://www.google.cn/maps/vt/lyrs=s@167) created by first author (J Chen), (**c**) registration result.

Figure 7 shows the registration result of a typical case with our method. Figure 7a,b show the CSOs extracted from the original image and the reference image respectively. 18 control points are obtained, and the matched CSOs and registration result is shown in Fig.7c.

### Comparative analysis of methods

In order to verify the effectiveness of our method, taking the level 18 online remote sensing imagery as the data source, 70 image pairs of the different area are cut in China from Tianditu, Google or BingMap respectively, and more than 8 objects within the overlapping area of the original image and the reference image are guaranteed. Then, these image pairs are used to carry out image registration experiments.

Since the registration method based on image grayscale is sensitive to rotation and deformation, and the method based on Fourier transform may have a large interpolation error, some feature-based registration methods, including SIFT, ORB, BRISK, AKAZE, and SuperPoint, are selected to compare with our method. For feature-based registration methods, their respective optimal parameters are determined experimentally, and the kNN matching algorithm is applied to get the reliable control points. In our method, the values of *α* and *β* are 0.8 and 7, respectively. Equation (15) is used for all conventional methods in image registration and the same filtering process as our method is applied to ensure the max distance error is less than *ε* or the number of control points is less than or equals to 3. The maximum power of polynomial equation is initially set to 2 for all methods and will be reduced to 1 if the number of available control points is less than 6.

To objectively evaluate the pixel error of image registration, each original image retains its spatial coordinates, and the pixel distance of the registered coordinates relative to the original coordinates is calculated pixel by pixel. The average pixel error (APE) of each registered image is calculated, and the number of image pairs with APE less than or equal to 5, 10, 15, 50 and greater than 50 are counted. The statistical results are shown in Table 6.

**Table 6:** Number of registered images with different average pixel error.

Method	APE (pixels)				
	≤ 5	≤ 10	≤ 15	≤ 50	> 50
SIFT	10	33	42	51	19
ORB	0	1	2	4	66
BRISK	10	21	24	28	42
AKAZE	15	31	39	44	26
SuperPoint	0	5	10	36	34
Our method	39	58	62	66	4

**Figure 8:**
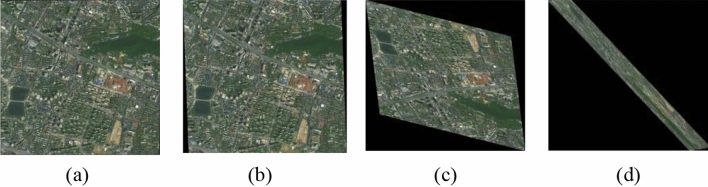
Examples of registered results of the cropped image from level 18 online Tianditu imagery (http://t0.tianditu.gov.cn/img_w/wmts)with different APE, created by first author (J Chen). (**a**) Our method with an APEof 3.35, (**b**) SuperPoint with an APE of 55.53, (**c**) BRISK with an APE of 1204.11, (**d**) ORB with an APE of 1696.75.

Figure 8 shows some registered results with different APE. As seen from the figure, the larger the APE, the worse the result of image registration. When the APE exceeds 50 pixels, the registered image has a large position deviation and even image distortion from the original image.

The case is considered a failed case in this paper when the APE value is greater than 15 pixels. The number of image pairs that can be successfully registered by different methods is counted according to this criterion. In order to objectively evaluate the registration accuracy of different algorithms, the failed cases are excluded and the minimum pixel error (MINPE), maximum pixel error (MAXPE) and average pixel error of different algorithms are statistically analyzed. Table 7 shows the statistical results.

**Table 7:** Accuracy comparison of different image registration methods.

Method	Success rate (%)	APE (pixels)	MINPE (pixels)	MAXPE (pixels)
SIFT	60	7.53	2.29	18.77
ORB	2.9	10.61	3.34	31.88
BRISK	34.3	6.07	1.37	17.53
AKAZE	55.7	6.63	2.39	13.78
SuperPoint	14.3	10.16	1.66	20.68
Our method	88.6	5.16	1.94	11.26

It can be seen from Table 7 that the sample cases with APE value less than 15 pixels account for 88.6% in our method, while the maximum proportion of the conventional methods is only 60.0%. Moreover, the registration error of our method is also smaller.

### Method adaptability analysis

#### The distributing density of CSOs

The distribution density of CSOs determines whether our method can be used in any area of cities. Taking partial areas of major cities in China as examples, 18-level Google online satellite images are used to extract sports fields, across-road bridges, across-river bridges, and urban intersections.

As shown in Table 8, the distribution density of extracted objects is more than 3 per km^2^ within the selected cities. The two images can be registered as long as the same coverage area of the two images is greater than 3km^2^ if *β* is set to 8 and the extraction error of Mask R-CNN is ignored.

**Table 8:** Number of characteristic spatial objects extracted in major cities of China.

City	SF	UI	AROB	ARIB	Total	Area (km^2^)	Density (km^-2^)
Fuzhou	276	1624	135	493	2528	710.41	3.56
Hangzhou	153	849	60	288	1350	319.13	4.23
Nanjing	202	630	63	112	1007	317.31	3.17
Wuhan	475	1636	211	509	2831	776.86	3.64
Changsha	179	879	105	103	1266	340.43	3.72
Guangzhou	698	2599	322	1689	5308	1683.34	3.15
Total	2038	8177	3434	3194	13,649	4147.48	3.29

The dispersiveness of object distribution in two images is also a factor to be considered in image registration, which determines the degree of local deformation. In order to analyze the distribution characteristics of the spatial objects extracted by Mask R-CNN, the experimental areas are evenly divided into small rectangular areas according to the side length, the percentage of the areas without objects, the average number, and the maximum number of CSOs contained in these rectangular areas are counted. Table 9 shows the object distribution under different side length of each rectangular area.

**Table 9:** Object distribution under different side length of rectangular area.

City	*λ* = 0.5 km			*λ* = 1 km			*λ* = 2 km		
	a (%)	b	c	a (%)	b	c	a (%)	b	c
Fuzhou	52.27	0.80	8	1.73	3.83	17	0.62	15.70	51
Hangzhou	12.48	1.10	13	0.61	4.40	25	0.00	19.80	78
Nanjing	22.01	0.86	10	1.71	3.52	25	0.00	15.84	56
Wuhan	40.80	1.00	9	7.68	4.00	18	0.74	16.60	76
Changsha	23.59	1.20	9	1.96	4.25	21	0.00	18.28	52
Guangzhou	36.50	0.96	14	4.42	3.74	27	0.00	14.59	75

*λ* the side length of each rectangular area, *a* the percentage of the areas without objects, *b* the average number of CSOs contained in rectangular areas, *c* the maximum number of CSOs contained in rectangular areas.

Table 9 displays that at least one object shall be ensured in the rectangular area when the side length of the rectangle is more than 1 km. There will theoretically be enough objects for image registration, and the control points are relatively evenly distributed in different areas of the image, if the overlapping area of two high-resolution images exceeds 9km^2^.

#### Analysis of the influence of spatial scale differences

In order to verify the influence of spatial scale differences of remote sensing images on image registration, an image successfully registered by all methods is selected from the above cases and is scaled in multiples of 0.25, 0.5, 0.75, 1.2, 1.5, 2.0, 4.0, 8.0 and 16.0. The new images are registered with the reference image respectively. The results are shown in Table 10.

**Table 10:** Results of Image registration at different spatial scales.

Method	Cell size of the resampled image (m)								
	0.15	0.3	0.45	0.72	0.9	1.2	2.4	4.8	9.6
SIFT	Y	Y	Y	Y	Y	Y	Y	Y	N
ORB	N	N	N	N	N	N	N	N	N
BRISK	N	N	Y	Y	Y	Y	Y	N	N
AKAZE	Y	N	Y	Y	Y	Y	Y	Y	N
SuperPoint	N	N	N	Y	N	N	N	Y	N
Our method	N	Y	Y	Y	Y	Y	N	N	N

*N* the failed case, *Y* the success case.

It can be seen from the table that both feature-based registration methods and our method have a suitable range of spatial scale. SIFT and AKAZE perform best among these algorithms in terms of spatial scale adaptation. Our method is applicable for spatial scale differences between 0.5 and 2 times.

## Discussion

Profit by the more stable CSOs, our method performs better success rate and spatial accuracy than conventional methods based on feature points. The method can be extended to the registration of HRRSIs in rural areas by adding rural characteristic spatial object categories. However, there still are a few problems to be solved.

The first challenge is to further improve the matching accuracy, which is closely related to the distribution density and dispersiveness of the objects extracted by Mask R-CNN on the image and the accuracy of positioning points. Although object distribution density and dispersiveness has been proved to be valid to some extent for our method in some cities. The number of available same objects will be further reduced, and the distribution dispersion may not be guaranteed due to the error of Mask R-CNN in extracting objects. This may increase the error of image registration, or even fail to complete the registration. On the other hand, the error of positioning points predicted by Mask R-CNN has a direct impact on the registration accuracy. There may be large errors in the positioning points for some spatial objects with complex shape, resulting in the decline of the quality of the control points.

Another problem that needs our attention is, the robustness of spatial scale, which is also a difficult problem faced by most image registration methods. Although the matching algorithm of control points has spatial scale independence, Mask R-CNN has a certain suitable scale range and will not be able to extract CSOs correctly beyond this range, resulting in registration failure. In order to improve the independence of spatial scale, it is necessary to establish a multi-scale model for extracting CSOs.

## Conclusions

In order to overcome the disadvantages of poor stability and reliability of local features, a method of automatic registration of urban HRRSIs based on CSOs is proposed. Typical urban artificial objects are selected as CSOs on the basis of the principles of being positionable, identifiable, ubiquitous, and stable, which are applied to study the automatic registration method of remote sensing images such as sports fields, across-road bridges, across-river bridges, and urban intersections. These objects and their positioning point are extracted from HRRSIs automatically by Mask R-CNN from the original image and the reference image respectively. The spatial relationship codes are also constructed according to the 28 nearest neighboring objects. The initial sequence of control points is screened by using the similarity of spatial relationship code. Then they are further screened through a position matching algorithm to obtain a relatively reliable sequence of control points. After substituting these control points into the polynomial equation and filtering out invalid ones, image matching is realized. Experimental results demonstrate that our method can register 88.6% of test images within a maximum average error of 15 pixels, compared to the maximum percentage of 60% for the best feature-based registration method (SIFT). On the other hand, the model also has quite robustness of spatial scale. When the spatial scale difference is within 0.5 and 2 times, the extracted CSOs can still be used for image registration. In future work, we will expand the categories of characteristic objects to ensure the dispersiveness of object distribution, build multi-scale model for extracting CSOs, and improve the accuracy of the positioning points. So that the performance of image registration of urban HRRSIs has been greatly improved based on CSOs.

## Data Availability

All data included in this study are available upon request by contact with the corresponding author.
